# Economic evaluation of artificial insemination of sex‐sorted semen on a Brown Swiss dairy farm—A case study

**DOI:** 10.1111/asj.13156

**Published:** 2019-02-10

**Authors:** Masahiro Osada, Hitomi Iwabuchi, Toru Aoki, Kika Sasaki, Hitoshi Ushijima, Takeyuki Ozawa

**Affiliations:** ^1^ Nippon Veterinary and Life Science University Musashino Tokyo Japan; ^2^ Tochigi Prefecture Ozasa Farm Tochigi Dairy Farmers Cooperative Nikko Tochigi Japan

**Keywords:** artificial insemination, Brown Swiss, dairy, farm management, sex‐sorted semen

## Abstract

Artificial insemination using sex‐sorted semen is employed to efficiently increase the number of female dairy calves born. Previous studies have determined that using sex‐sorted semen is beneficial to improve the management, but the mechanism by which it increases cattle numbers through objective indices of breeding remains unclear. This study focused on a Brown Swiss cattle herd in which frozen female sex‐sorted semen was systematically employed to increase the number of cattle. We analyzed the correlation between the increase in the number of cattle and the screening accuracy of sex‐sorted semen, measuring indices such as pregnancy rate and birth rate of female calves. Study revealed that: (1) production cost for female calves is influenced by the pregnancy rate, rate of female calves, and using sex‐sorted semen is less expensive than using nonsorted semen; (2) improvements in screening accuracy nearly doubled the number of cows and tripled the number of heifers in 5 years; and (3) use of sex‐sorted semen improved milk quality. The pregnancy rate was lower when sex‐sorted semen was used, but the birth rate of heifers was improved. Results suggest that artificial insemination using sex‐sorted semen is beneficial because it economically produces offspring to increase the herd.

## 1 INTRODUCTION

**Figure 1:**
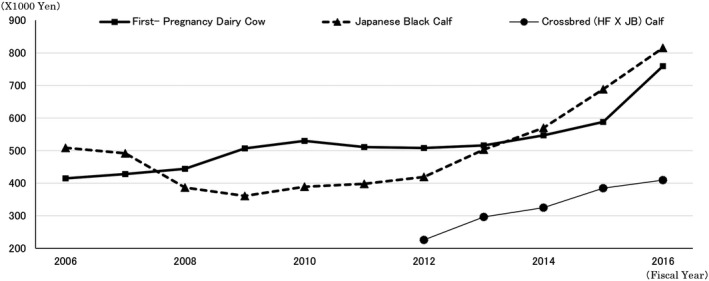
Changes in price for first pregnancy cows, beef and dairy cross bred calves. *Source*: Agriculture and Livestock Industries and the Hokuren Federation of Agriculture Cooperatives

Japan has introduced policies to prioritize efficient breeding methods and high productivity in commercial dairy farms, and the number of cattle has rapidly expanded to 80.7 individuals per farm (Arai, 1989; Ministry of Agriculture, Forestry and Fisheries, 2017). Meanwhile, as feeder calf prices have soared, dairy farmers in many prefectures have begun to choose hybrid production, using semen from Japanese Black cattle rather than dairy cattle semen in artificial insemination or producing Wagyu though embryo transfer (Osada, Ushijima, & Ozawa, 2017). Although offspring production of dairy cattle has been guaranteed, the price of first‐pregnancy dairy cows in Hokkaido has skyrocketed along with the price of beef cattle, with first‐pregnancy dairy cows regularly commanding prices in excess of 1 million yen per head in 2017 (Ozawa, Lopez‐Villalobos, & Blair, 2005). There is concern that the current guaranteed state of offspring will worsen as trends such as individual and cooperative farming become more popular (Osada, Obuchi, Ushijima, & Ozawa, 2013; Osada, Ushijima, & Ozawa, 2015) (Figure 1).

**Figure 2:**
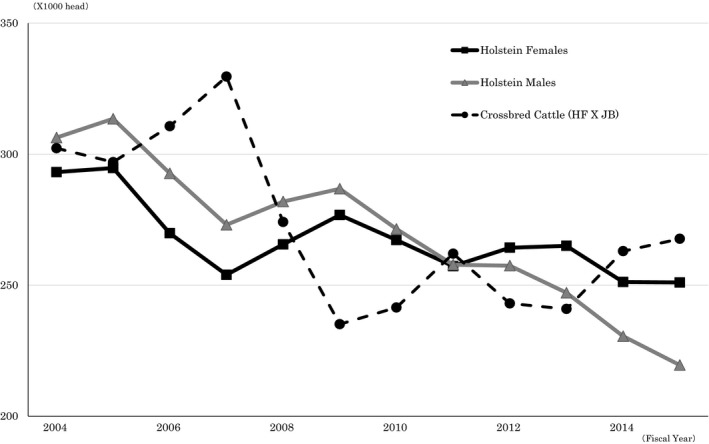
Nationwide changes in births by type. *Source*: Livestock Breeding Center

Securing guaranteed offspring is an important aspect of dairy farming, and the use of sex sorting technology for semen has prompted a number of studies of efficient methods for securing heifer calves (Hamano, 2015; Kataoka et al., 2015; Kawano, Toma, & Sembokuya, 2014; Obuchi, Osada, Ushijima, & Ozawa, 2013). The utilization of domestic sex‐sorted semen has increased to 12.9% (AIAJ, 2017), and its effectiveness has been confirmed, as the birth rate of female calves exceeded that of bull calves in 2015 (Figure 2). Osada (2017) noted that the intended use of female sex‐sorted semen is not to increase the overall number of female cows nationwide, but rather to secure the necessary number of female calves to maintain personal herds through efficient heifer calf production. Therefore, it is possible to receive tax exemptions on profits from beef sales in industries aimed at increasing the numbers of first‐pregnancy heifers. These exemptions have expanded from dairy cattle sales to industries raising cattle for beef, including the production of crossbreeds and selective transfer of Japanese Black embryo. Although sex screening technologies have advanced from the diffusion stage to the practice/effect verification stage, to date there is no analysis of the process by which this technology affects the production of female calves, through objective indices of breeding technology.

At the 193rd Ordinary Diet session, draft legislation was revised to amend part of the law concerning livestock management stability. As of 2018, Japan will abolish the provisional aspect of laws such as the Act on Temporary Measures Concerning Compensation Price for Producers of Milk for Manufacturing Use, making the system permanent, and will expand the targets for tariff elimination restricting partial consignments and supplementary milk producers in order to promote distinctive dairy farming systems. As part of these revisions, a “premium trading system” was introduced in 1998 focusing on the elasticity of raw milk consignment sales, enabling farmers to negotiate prices for individual collections of distinctive raw milk or to add premiums to regular milk prices. The distinctive raw milk produced by Brown Swiss cattle is sold as milk and as other dairy products. As the size of herds has increased in recent years, so have expectations for boosting the diversity of raw milk trading.

This study focuses on cases where female sex‐sorted semen was used to increase the production of Brown Swiss cattle (Endo, Kuroki, & Tanaka, 2017), in order to better understand the mechanism by which female sex‐sorted semen affects the increase in cattle population in terms of objective indices of breeding technology. In addition, we consider the guaranteed offspring policy associated with using sex selection technology, and consider its implications for sustainable dairy farming.

## 2 MATERIALS AND METHODS

**Figure 3:**
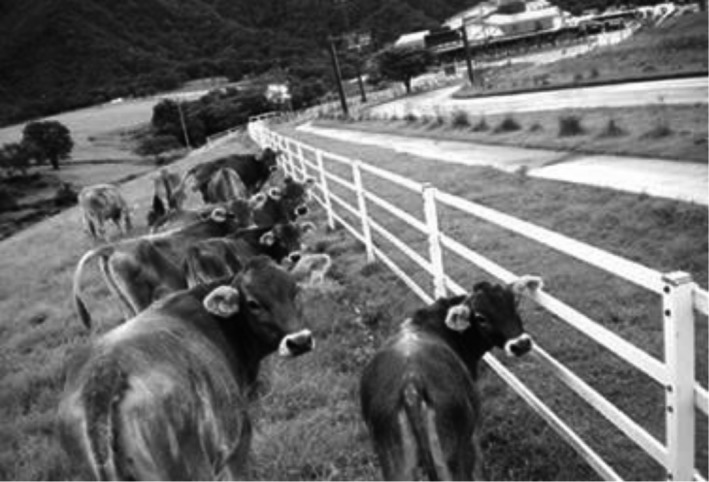
Nikko Kirifuri‐kogen Ozasa Farm (Resthouse in Background)

The Ozasa Farm Dairy Farming Cooperative in Tochigi prefecture is located at an altitude of 1,300 m, at the northern end of Nikko Kirifuri highland, facing the Nikko Mountains. In the summer of 1957, 112 ha of private land was purchased by members of the cooperative, to be used as a breeding farm open to public use (Figure 3). Later, additional pasture was purchased from the state and from private owners; today, the farm contains approximately 300 breeding dairy cows and a grazing area of 362 ha. In 1976, the Ozasa Farm Resthouse was opened, and in 1998, processing facilities were expanded to increase dairy product production and sales. The cooperative is also working on a “food experience studio,” to educate the public about food.

In 1998, 20 head of Brown Swiss cattle from the United States were introduced. The farm has a goal of increasing the herd through the use of frozen female sex‐sorted semen; currently 110 cattle are being reared. A summary of the business and production technology being used was compiled from official cattle test results by Livestock Improvement Association of Japan, interviews, and reproductive technology indicators such as the frequency of insemination using frozen female sex‐sorted semen and pregnancy rates extracted from individual breeding control registers from 2013 to 2017. Furthermore, the insemination rates of frozen female sex‐sorted semen and nonsorted semen were compared, female production efficiency was calculated based on the sex of offspring, and the effect of using female sex‐sorted semen was analyzed. Also, heifers were younger than 14‐month‐old nonpregnant cows, and were over 14‐month‐old pregnant cows.

## 3 RESULTS AND DISCUSSION

### 3.1 Fertility of female sperm

Changes in the number of and pregnancy rate for heifers/dairy cows according to type are indicated in Table 1. Although the use of frozen female sex‐sorted semen fluctuates from year to year, from 2013 to 2016, artificial insemination using frozen female sex‐sorted semen was used 231 times, a utilization rate of 49.3%. The pregnancy rate from female sex‐sorted semen was 83/231 inseminations (35.9%); the rates for dairy cows and heifers were 32.4% and 41.6%, respectively, with heifers 9.2 points higher, which is not significantly different from the results of previous studies (Nakao, 2005). Studies have noted that the number of heifer calves produced increases with specific use of sex selection technology (Kataoka et al., 2015), and as heifer pregnancy rates are higher than dairy cow pregnancy rates, specific use of sex selection technology is particularly recommended for heifers. Although the use of female sex‐sorted semen does not increase the rate of abnormalities (*teratogenicity)*, the sperm survive only a short time, and the concentration of sperm is typically 3,000,000–6,000,000 sperm per ejaculate (less than one tenth of nonsorted semen), which is said to contribute to lower conception rates (Karakaya et al., 2014).

**Table 1:** Brown swiss frozen semen conception rate 2013–2016

Use of total sex‐sorted semen		Heifers				Female cows				Total				
		Female sex‐sorted semen		Non‐sorted semen		Female sex‐sorted semen		Non‐sorted semen		Female sex‐sorted semen		Non‐sorted semen		Total
Year	%	%	Count	%	Count	%	Count	%	Count	%	Count	%	Count	%
2013	63.5	52.4	21.0	62.5	8.0	50.0	26.0	36.8	19.0	51.1	47.0	44.4	27.0	48.6
2014	64.6	36.8	19.0	33.3	6.0	22.2	54.0	44.1	34.0	26.0	73.0	42.5	40.0	31.9
2015	49.0	41.7	36.0	65.2	23.0	30.6	36.0	28.8	52.0	36.1	72.0	40.0	75.0	38.1
2016	28.9	30.8	13.0	38.9	36.0	38.5	26.0	50.0	60.0	35.9	39.0	45.8	96.0	43.0
Total	51.5	40.4	22.3	50.0	18.3	35.3	35.5	39.9	41.3	37.3	57.8	43.2	59.5	40.4

*Note*. In case of multiple artificial insemination sessions per cycle, the number was recorded as “once.”

### 3.2 The cost of producing female offspring

The cost to produce a female calf using frozen female sex‐sorted semen was calculated, taking into consideration the results of using sex‐sorted semen and pregnancy rates. The cost is based on a number of variable factors, such as the price of artificial insemination, the price of frozen sex‐sorted semen, the pregnancy rate, and the female production ratio. Therefore, the expenses were calculated using the formula (compound rate + raw material)/rate of pregnancy/female probability (Table 2). This price represents the production cost of a single female calf and does not include the loss of milk revenue due to declining pregnancy rates or expenses incurred from prolonged fertility treatments. The rate of female calf production from nonsorted semen is approximately 48% (Hamano, 2015); in the case of artificial insemination, with a pregnancy rate of 40%, the cost of a female calf equals 46,875 yen. The pregnancy rate when female sex‐sorted semen is inseminated is 10% lower; however, the female production rate is 90%, bringing the price per female calf to 44,444 yen. Thus, the use of female sex‐sorted semen is an efficient method of producing female offspring.

**Table 2:** Estimated production cost per female offspring in 2016

Division		Compound rate	Raw material	Rate of pregnancy (%)	Female offspring probability (%)	Cost (Yen)
Trial calculation	Nonsorted semen	3,000	6,000	40.0	48.0	46,875
	Female‐sexed semen	3,000	9,000	30.0	90.0	44,444
	Imported female‐sexed semen	3,000	9,000	30.0	80.0	50,000
2013–2016 Ozasa farm	Nonsorted semen	3,000	6,000	43.3	53.5	38,851
	Female‐sexed semen	3,000	9,000	35.9	73.8	45,293
	Sort 90 trial calculation^a^	3,000	9,000	35.9	90.0	37,140

*Note*. It is regarded as a synonym for domestic distributed sex‐sorted frozen semen among Japanese dairy farmers.

Source: Interview publication, 2016.

a Sort 90 is the private “Semen sexing technology” produced by Livestock Improvement Association of Japan (LIAJ).

Of note, all female sex‐sorted semen of Brown Swiss on the market in 2015 was imported, and the sorting accuracy of imported semen may be less than 90% (Ushijima, Geshi, Akagi, Izaike, & Yoshiaki, 2018). If the probability of producing female offspring from imported sorted semen is only 80%, each female calf produced will cost 50,000 yen, making the process less cost‐effective for dairy farmers.

Production costs per female calf were estimated based on the pregnancy rates of Ozasa Farm between 2013 and 2016, and from the female production rate of both nonsorted frozen semen and frozen female sex‐sorted semen. With regard to the pregnancy rate of nonsorted semen, 2015 appeared to be an outlier with a 100% pregnancy rate, much higher than the average 53.5%, but the accuracy of female sex‐sorted semen was 73.8%, unusually low. Based on 2016 pregnancy rates and the probability of producing female offspring, the price per head for nonsorted semen fertilization is 38,851 yen, and that for female sex‐sorted semen is 45,293 yen, which is higher. However, the rate of production of female calves cannot be determined by the dairy farms, and responsibility lies solely with semen suppliers. As current frozen female sex‐sorted semen on the market must meet a screening purity rate of 90% or more, the rate of female offspring produced should be 90%. In the trial calculation for average pregnancies from 2013 to 2016, the rate was 35.9%, indicating a cost of 37,140 yen per cow. The cost of producing calves using frozen female sex‐sorted semen with a low pregnancy rate is equivalent to the rate of production using nonsorted frozen semen.

### 3.3 Improvements to cattle production and breeding technologies

**Figure 4:**
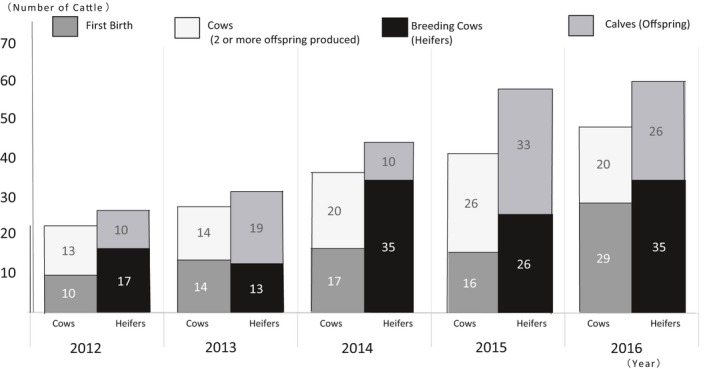
Changes to livestock numbers by type. *Source*: Interview Publication

Figure 4 and Table 3 both show Ozasa Farm cattle herd breeding trends according to type. From 2012 to 2016, neither Japanese Black semen nor transfer of Japanese Black embryos was used for feeder calf production, and all artificial insemination used imported Brown Swiss semen. Of the 48 female calves produced in 2014, eight were conceived through embryo transfer. In order to conduct embryo transfer, 10 Holstein heifers were externally inseminated. The pregnancy rate for frozen sex‐sorted embryos was 64.3% (9 pregnancies/14 transfers), increasing the cattle count 2.2‐fold.

**Table 3:** Changes in management operations

Year	Breeding count (per head %)				Breeding head count average growth rate (%)	Number of female offspring	Selected offspring
	Cows	Growth rate	Heifers	Growth rate			
2012	23	—	27	—	—	20	—
2013	28	21.7	32	18.5	20.0	17	6
2014	37	32.1	45	40.6	36.7	48	5
2015	42	24.3	58	28.9	22.0	28	5
2016	49	6.5	61	5.2	10.0	37	15
Average	35.8	21.2	44.6	23.3	22.2	30.0	7.8

*Note*. Source: Ozasa Farm Herd Examination Results and Individual Breeding Control Registers.

The use of frozen female sex‐sorted semen with artificial insemination in 2013 led to an increase in the number of heifers prior to the 2014 inseminations, with utilization rates of frozen female sex‐sorted semen reaching as high as 64.6%. In 2014, the use of frozen female sex‐sorted semen for second and subsequent pregnancies rose to 45.9%, with a decline in overall pregnancy rates to 31.9%. As for the increased number of calves conceived in 2015, while there were slight improvements due to the use of female sex‐sorted semen, pregnancy rates were as low as 38.1%. According to these results, the factors that determine pregnancy rate are the number of heifers who were unable to conceive in the previous year and the utilization rate of female sex‐sorted semen that year, as well as the female sex‐sorted semen fertilization rate for inseminations occurring two or more times (Table 4).

**Table 4:** Factors affecting conception rate and precision of female‐sexed semen

		2012		2013		2014		2015		2016	
		23.3		48.6		31.9		38.1		43.0	
Rate of conception(%)	Average	%	Factor	%	Factor	%	Factor	%	Factor	%	Factor
Heifer rate (nonconception)	56.3	63.0	**↑**	40.6	**↓**	77.8	**↑**	44.1	**↓**	57.4	**→**
Use of female‐sexed semen	50.3	―	―	63.5	**↓**	64.6	**↓**	49.0	**→**	28.9	**↑**
Insemination with female‐sexed semen 2 or more times (%)	38.3	―	―	23.3	**↑**	45.9	**↓**	41.5	**→**	40.0	**→**
Semen accuracy		%	Factor	%	Head	%	Head	%	Head	%	Head
Number of births from use of sex‐sorted Semen (number of female calves produced)	73.8	―		44.4	9(4)	78.3	23(18)	73.3	15(11)	83.3	18(15)
Number of births from nonsorted semen (number of female calves produced)	53.5	―		100.0	6(6)	63.6	11(7)	36.4	11(4)	40.0	15(6)
Rate of female calves birthed (%)	65.7	―		66.7		73.5		57.7		63.6	

*Note*. The average rate of nonfertile cattle is 56.3%, and the average sex‐selected semen utilization rate is 50.3%.

Source: Ozasa Farm Individual Breeding Control Registers.

We must endeavor to improve the rate of conception using frozen female sex‐sorted semen by paying attention to the method of insemination and semen characteristics. In an analysis that simulated an increase in the use of sex selection technology, when frozen female sex‐sorted semen was used under conditions of low conception rate, it became difficult to guarantee offspring, leading to a reduction in profitability due to the increase in operational expenses and delays in conception rates. For the strategy to be effective, the conception rate must be at least 45%, and female sex‐sorted semen must be used at least 50% of the time (Kawano et al., 2014).

The pregnancy rate for nonsorted semen at Ozasa Farm is 45% or lower. While the rate of pregnancy resulting from at least two uses of female sex‐sorted semen has gradually decreased from its 2014 peak of 45.9%, the utilization rate exceeds 60%, from which it can be inferred that the rate of pregnancy has decreased. As pointed out in previous simulation analyses, the pregnancy rate deteriorated and reproductive performance worsened, with issues such as an increase in the number of empty placentas or an increase to the number of dry days. In response to these problems, the use of female sex‐sorted semen was reduced after 2015, in an effort to improve pregnancy rates. Although the decline in conception rate is a concern, the average number of female calves produced between 2013 and 2016 was 30, with a female production rate of 65.7% and a replacement rate of 45.5%. Thus, female offspring were efficiently secured using female sex‐sorted semen.

It remains problematic that technical indicators related to the breeding of Brown Swiss cattle showed low values for all items, excluding average production order and update rate, compared with national management technology indicators and 2016 performance results obtained by dairy cattle groups (Table 5). As the replacement rate is influenced by the number of newly lactating dairy cows, farmers who wish to increase the size of their herd should focus on increasing the replacement rate; however, our data showed a decrease in the average number of calves produced. Calving intervals increased more than 20% due to a decline in the pregnancy rate and increases in the number of open days, the number of dry days, and the number of insemination attempts required to conceive. For example, the typical interval of 151 empty days was extended 31.8 days by farmers according to technology indices, highlighting the difficulties that farmers experience with breeding and keeping these cattle compared with Holsteins.

**Table 5:** Changes in technology indices

Year	Calving interval (days)	Days open	Length of dry period (days)	Number of times required to conceive^a^	Number of days after birth for initial insemination	Average age (months)	Average number of calves produced	Update rate (%)^b^
2012	533	131.7	44.0	1.4	107.9	63.5	2.9	–
2013	442	169.8	125.3	2.6	95.2	51.9	2.0	47.8
2014	461	155.9	163.7	2.8	75.1	51.0	2.0	50.0
2015	462	182.0	267.0	2.5	70.9	54.0	2.2	27.0
2016	532	191.5	155.1	2.6	80.4	48.9	1.8	57.1
Average	486	166.8	151.0	2.4	85.9	53.9	2.2	45.5
Technology Indices^c^ (2016 Bovine Group Test Results)	426 (432)	135	60 (65)	1.8	―	―	2.8	23

Notes

a Calculated by the number of conceptions/total insemination number, including heifers.

b Renewal rate calculated by the new number of cattle in a year/the number of cattle at the beginning of the year.

c Technical indices are for Holsteins.

Source: Ozasa Farm Individual Breeding Control Registers.

In 2012, a plan was devised to improve breeding‐related traits and milk quality by eliminating cattle that were unable to conceive for long periods of time and older cattle. Starting in 2013, female sex‐sorted semen was used. In 2016, attempts were made to improve milk quality through planned selection; as a result, there was a decrease in the per dairy cow milk yield, correlated with an increase in the number of first‐time calves. Improvements in breeding efforts were attempted, resulting in an increase in milk production but a decrease in the number of somatic cells per 108 thousand units/ml (Table 6). This approves improvement of milk quality with disadvantage of milk yield. The increase in the number of breeding cows since 2014 is remarkable, with an increased replacement rate of 45.5% in 2016. Even though the pregnancy rate is around 40%, the increase in the number of female calf births is directly related to the active use of female sex‐sorted semen, which has risen to 60% (Kataoka et al., 2015).

**Table 6:** Trends in production technology indicators

	Milk production per cow (kg)	Milk fat (%)	MFSF rate (%)	Somatic cells (10,000 units/ml)
2015	6,492	4.09	9.39	299
2016	6,082	4.36	9.51	108

*Note*. Source: Ozasa Farm Cattle Test Results.

In an effort to expand processing and sales, Ozasa Farm achieved a female delivery rate of 65.7% after 4 years of thorough use of sex‐sorted insemination techniques, increasing the number of cattle in the herd. Both herd replacement rate and milk quality improved, making more high‐quality milk and dairy products available to consumers, and demonstrating a successful sustainable dairy farming model. Utilization of sex screening technology was confirmed to be an economical and efficient strategy for in‐farm production of cattle while increasing the size of the herd (Osada et al., 2013). However, when using frozen female sex‐sorted semen, it is necessary to maintain cattle rearing standards so that the rate of pregnancy does not decrease. Furthermore, it is necessary to properly respond to issues related to rearing facilities and labor during rapid increases in the cattle population. Breeding improvements should be considered under the cattle renewal rate/possible population increase plan, while farmers who are not interested in securing successor cattle must be able to earn high profits using transfer of Japanese Black embryos.

Meanwhile, as many prefectures do not have an environment conducive for producing calves or raising breeding cattle, cattle breeding training programs should be provided to areas that have ceased production and collective farms with milking stations should be rebuilt. Policy assistance should be provided to enable farmers to use sex selection technologies that can guarantee offspring production, as this technology enables sustainable modern dairy farming.

## ACKNOWLEDGMENTS

This research was conducted with the help of the Grants‐in‐Aid for Scientific Research (C) (Issue Number: 16X07910), a study of favorable measures to guarantee offspring and development conditions for public breeding farms.
